# The Impact of Latent Cytomegalovirus Infection on Spontaneous Abortion History and Pregnancy Outcomes in Romanian Pregnant Women

**DOI:** 10.3390/microorganisms12040731

**Published:** 2024-04-03

**Authors:** Adelina Geanina Mocanu, Dana Liana Stoian, Ana-Maria Cristina Daescu, Alexandru Catalin Motofelea, Ioana Mihaela Ciohat, Dan Bogdan Navolan, Tatjana Vilibic-Cavlek, Maja Bogdanic, Dragos Nemescu, Larisa Tomescu, Adrian Carabineanu

**Affiliations:** 1 Department of Obstetrics-Gynecology, Victor Babes University of Medicine and Pharmacy Timisoara, P-ta Eftimie Murgu nr. 2, 300041 Timisoara, Romania; adelina.mocanu@umft.ro (A.G.M.); tomescu.larisa@umft.ro (L.T.); 2 Doctoral School, Victor Babes University of Medicine and Pharmacy Timisoara, P-ta Eftimie Murgu nr. 2, 300041 Timisoara, Romania; 3 Department of Endocrinology, Victor Babes University of Medicine and Pharmacy Timisoara, P-ta Eftimie Murgu nr. 2, 300041 Timisoara, Romania; stoian.dana@umft.ro; 4 Department of Psychiatry, Victor Babes University of Medicine and Pharmacy Timisoara, P-ta Eftimie Murgu nr. 2, 300041 Timisoara, Romania; ana-maria.daescu@umft.ro; 5 Department of Internal Medicine, Victor Babes University of Medicine and Pharmacy Timisoara, P-ta Eftimie Murgu nr. 2, 300041 Timisoara, Romania; alexandru.motofelea@umft.ro; 6 Laboratory of Antenatal Medicine, Timisoara City Emergency Hospital, 300202 Timisoara, Romania; ioana.ciohat@umft.ro; 7 Department of Virology, Croatian Institute of Public Health, 10000 Zagreb, Croatia; maja.bogdanic@hzjz.hr; 8 School of Medicine, University of Zagreb, 10000 Zagreb, Croatia; 9 Department of Obstetrics-Gynecology, Gr. T. Popa University of Medicine and Pharmacy Iasi, Universitatiistr. nr. 16, 700115 Iasi, Romania; dragos.nemescu@umfiasi.ro; 10 Department of Surgery, Victor Babes University of Medicine and Pharmacy Timisoara, P-ta Eftimie Murgu nr. 2, 300041 Timisoara, Romania; carabineanu.adrian@umft.ro

**Keywords:** latent cytomegalovirus, spontaneous abortion, pregnancy complications, neonate

## Abstract

Cytomegalovirus (CMV), a DNA virus that belongs to the *Orthoherpesviridae* family, infects 40–100% of people. Primary/non-primary CMV infection during pregnancy could cause fetal disabilities. After primary infection, CMV causes a latent infection and resides in cells of the myeloid compartment (CD34^+^, monocytes). Few studies have analyzed the impact of latent CMV infections on miscarriage history, pregnancy complications, and neonatal outcomes. Methods: Serum samples from 806 pregnant women (28.29 ± 4.50 years old) who came for a consultation at the Timisoara Clinical Emergency City Hospital between 2008 and 2010 were tested for anti-CMV IgM/IgG antibodies, and data about demography, obstetrical history, pregnancy complications, birth, and neonate were collected. The data were compared between the groups with and without latent CMV infection, and statistical significance was calculated. Results: We did not find a difference regarding cesarean section (OR = 0.916, *p* = 0.856), placental abruption (OR = 1.004, *p* = 1.00), pregnancy-induced hypertension rate (OR = 1.078, *p* = 1.00), secondary sex ratio (0.882, *p* = 0.857), APGAR score (*p* = 0.225), gestational age at birth (*p* = 0.434), or birth weight (*p* = 0.365). A borderline significant difference was found regarding the presence of miscarriage history: OR = 8.467, *p* = 0.051. Conclusions: The presence of latent CMV infection does not affect the likelihood of complications in healthy women. A borderline significantly higher prevalence of miscarriage history was found in women with latent CMV infection.

## 1. Introduction

Cytomegalovirus (CMV), a DNA virus that belongs to the *Orthoherpesviridae* family, is widespread among humans; a percentage of between 40% and 100% of people are infected with CMV worldwide [1]. The fact that two national institutions in the USA, the National Vaccine Advisory Board and the Institute of Medicine, have recommended developing a CMV vaccine for intensive research highlights the socioeconomic burden of CMV infection [2,3]. The prevalence of CMV infection varies depending on many factors, such as age, geographic region, occupation, and socioeconomic status [4]. Studies show that the prevalence increases with age; thus, in the USA, it ranges between 60% at the age of 40 and 90% at the age of 80 [5]. In other parts of the world, such as developing countries with poor sanitation, the prevalence of CMV infection may be higher [4]. Studies performed in Romania showed that the CMV prevalence decreases with increasing hygienic measures [4].

CMV infection is transmitted between humans by contact with body fluids from an infected individual (saliva, blood, urine, semen, breast milk, tears, and vaginal fluids) [6]. Symptoms of CMV infection vary from asymptomatic infection to mononucleosis-like symptoms (fever, malaise, headache, and fatigue) or more severe symptoms in people with affected immune response (HIV/AIDS, immunosuppressed graft recipients, cancer patients under chemotherapy, or newborns) [1,7,8].

The main concern is whether a primary CMV infection occurs in pregnant women, because the virus could pass through the placenta and affect the fetus, causing disabilities such as hearing or vision loss, intellectual and neuronal impairment, etc. [9]. Although antiviral therapy and specific immunoglobulins have been tested to reduce the risk of fetal damage, the best prevention is reducing the risk of infection [10,11]. Pregnant women should avoid contact with the bodily fluids of young children and maintain good personal hygiene.

Most newborns with congenital CMV infection are born following a maternal CMV non-primary infection (reinfection or reactivation). Also, previous studies show that the possibility of “ping-pong” infections between infants and mothers exists, since infants often acquire CMV through breast milk, while mothers acquire infection after primary infection in their infants [12]. Another factor that allows reinfection is the immunodeficient status of the pregnant woman [13,14].

To intercept women at risk for primary infection in pregnancy, many countries include serological screening for CMV in their care schedule [4,15,16,17]. The rationale for testing CMV immune status is based on the fact that anti-CMV IgG seronegative women who are at risk for primary infection should be counseled to avoid contact with CMV-infected people [18]. Fetuses of anti-CMV IgG-seropositive pregnant women can also be infected due to a non-primary infection caused by reactivation or infection of pregnant women with another viral strain [18]. However, in this latter situation, the symptoms are less severe [18,19,20], and the risk of vertical transmission is much lower (1–3%) compared to primary infection (30–40%) during pregnancy [21].

Previous research reveals the life cycle of CMV and the mechanisms to establish latency [22,23]. CMV can infect a broad range of cells, including muscle cells, fibroblasts, immune cells (macrophages, dendritic cells, etc.), hepatocytes, and endothelial cells [22,24]. Similar to *Toxoplasma gondii*, CMV causes a lifelong latent infection that occurs after primary infection [15]. The cellular reservoirs of CMV, where the virus remains silent in the latent phase, are the myeloid cells of the bone marrow, including CD34^+^ hematopoietic progenitor cells and circulating monocytes [24]. In certain conditions, CMV could undergo reactivation cycles [25]. Reactivations of latent CMV occur more often in immunocompromised individuals (graft recipients, HIV-infected people, immunosuppressed people, etc.) than in healthy people [26,27]. After primary infection, the latent CMV infection induces changes in the host’s immune system at the level of T lymphocytes [28], monocyte innate immune response [29,30], cytokine production, [31] and in several tissues, such as placenta veins [32], blood clot formation [33], HLA-G molecule expression [34], and vessel walls [35]. As most of these biomarkers were found to be associated to some degree with pregnancy complications in recent studies, we could speculate that latent CMV infection influencing the level of some biomarkers could also have an impact on the occurrence of certain pregnancy complications. Thus, T lymphocytes are involved in tolerance induction in recurrent pregnancy loss [36], and monocyte immunomarkers are associated with preterm birth [37,38,39], changes in HLA-G expression with pregnancy loss [40,41], and clot formation and infarction in placenta with intrauterine growth restriction (IUGR) [42].

CMV primary and non-primary infections in pregnant women represent important health issues because congenital CMV infection may lead to neurosensorial hearing loss and other fetal disabilities. However, there is no consensus regarding screening for CMV infection in pregnant women; some countries recommend it [4] and others do not [43]. Vertical transmission is higher in primary than in non-primary infection [21] and in the third trimester compared to the first trimester of pregnancy [43]. The severity of fetal lesions and long-term disabilities is higher after first-trimester congenital infection [43] Studies from pregnant women and umbilical cord plasma showed the presence of CMV genome [44]. In some cases, CMV was detected consistently over time in the plasma of certain women, and this could argue for the free circulation of reactivated virus [44,45]. All these above-mentioned observations support the role of screening against CMV infection in pregnant women and the implementation of hygienic measures to reduce congenital infection [43].

Most studies analyzed the effect of a primary CMV infection throughout the course of pregnancy or on the fetus [46,47]. Only a few studies describe the impact of latent CMV infection [48,49]. These latter studies analyzed the association of latent CMV infection with the occurrence of recurrent pregnancy loss [50,51,52,53,54] and the prevalence of pregnancy complication such as HELLP syndrome [55], preeclampsia [55,56,57,58,59,60,61,62,63,64,65,66,67], fetal death [68], and low birth weight [68]. The results regarding the association between latent CMV infection and obstetrical outcome showed contradictory results.

Considering the above, this study aimed to analyze the association between latent CMV infection and demographic features, miscarriage history, pregnancy, and neonatal outcomes in pregnant women. We ran our research on a reliable database in which the gestational age was established to within an accuracy of three or five days based on first- or second-trimester pregnancy, respectively [69,70]. This study is part of a larger study that analyzes the epidemiology of TORCH agents in the Western region of Romania [4,71,72,73], and the impact of latent CMV and *Toxoplasma gondii* infections on the fetus and pregnancy outcome [15].

## 2. Materials and Methods

### 2.1. Study Design, Population, and Setting

A retrospective study was conducted from 2008 to 2010 at the Emergency Clinical City Hospital in Timişoara, Romania, and included 806 pregnant women who had a residence in any of three counties (Timis, Caras-Severin, and Arad) situated in the western region of Romania. The pregnant women were tested between 4 and 12 weeks of gestation against IgG-/IgM- anti-*Toxoplasma gondii*, anti-CMV, and anti-rubella virus antibodies, and were included in the study based on a consecutive case population and their arrival.

Only pregnant women who tested negative for anti-*Toxoplasma gondii*, anti-CMV, and anti-rubella IgM antibodies were included in the study. The pregnant women who were seropositive for IgM-anti-*Toxoplasma gondii*, CMV, or rubella virus in a screening test were retested by a second serological test and were excluded from the study.

At the time of blood sampling, participants were asked about demographic data [year of birth, age, place of residence (urban, rural)]. Later, after birth, data about the presence of pregnancy complications (pregnancy-induced hypertensive disease, premature spontaneous rupture of membranes, placental abruption, abnormal placental insertion, etc.), mode of the establishment of gestational age, gestational age at birth, and mode of delivery were collected from medical files for each pregnant woman. Also, data about the newborn (weight at birth, gender, Apgar score, length, and head circumference) were identified and added to the data collected at the time of blood sampling. The gestational age was established by ultrasound measurements (first-trimester crown–rump length or second-trimester composed ultrasound) for all pregnant women, as published before and reported in our previous publication [14]. Among the 806 pregnant women with all available data, 336 women gave birth by cesarean section birth and 470 by vaginal birth. We evaluated the impact of latent CMV infection on pregnancy duration in 470 pregnant women with vaginal birth. The reason for the exclusion of pregnant women who gave birth by cesarean section was that cesarean section is an iatrogenic procedure performed often at a time different from the moment of spontaneous labor occurrence.

The deviation between the weight at birth and the expected weight at birth for each newborn was estimated and the MoM (multiple of medians) was calculated by dividing weight at birth by the expected weight. We used the nomograms developed by Talge et al. to define the expected birth weight of the neonates according to gestational age and gender [74]. To analyze the impact of latent CMV infection on the newborn’s weight, we calculated a MoM for each newborn, and the MoMs were compared for the newborns in the two groups: with and without latent CMV infection.

### 2.2. Detection of IgG Antibodies against Toxoplasma gondii, CMV, and Rubella Virus

Measurement of IgG and IgM anti-*Toxoplasma gondii*, CMV, and the anti-rubella-virus antibody titer was performed by the chemiluminescence method using an Immulite One Machine and commercial tests (Siemens Healthcare Diagnostics Products, Marburg, Germany). IgM-positive samples were retested by enzyme immunoassay (Roche Diagnostics, Basel, Switzerland). According to cut-off values recommended by the manufacturer, serological results were grouped into positive, negative, and inconclusive values. For statistical analysis, cases of inconclusive results were considered along with seronegative (non-immune) cases due to the uncertainty of immunization.

### 2.3. Statistical Analysis

We used the Astraia (Version 1.22.1) maternofetal module (Astraia GmbH, Munich, Germany) and Microsoft Office Excel 2021 (Redmond, WA, USA) to store data. SPSS version 26 (SPSS Inc, Chicago, IL, USA) was used for statistical analysis. Data are presented as percentages for categorical variables, or medians, and interquartile ranges (IQRs) for continuous variables without Gaussian distribution. The value of significance of the differences between groups was assessed using the Mann–Whitney U test or Kruskal–Wallis (medians, non-Gaussian populations) and χ^2^ (proportions) tests, as previously published [15]. Continuous-variable distributions were tested for normality using the Shapiro–Wilk test and for equality of variance using Levene’s test. Sample-size calculations were performed before the study, aiming to provide a confidence level of 95%. In this study, *p* < 0.05 was considered the threshold for statistical significance. 

We analyzed the association between the presence of the latent CMV infection and the history of spontaneous abortion (SA) in two ways. Firstly, we counted the number of women with spontaneous abortion (total 84) and compared it with the number of women with at least one previous pregnancy (gestation ≥ 2, total 465) in the groups of women with and without latent CMV infection. Secondly, we counted the total number of spontaneous abortions (total 108) and compared it with the number of all pregnancies (total 828) that the women (gestation ≥ 2) had before in the two groups with and without latent CMV infection.

## 3. Results

### 3.1. Demographic and Clinical Features of Pregnant Women Included in the Study

The pregnant women (n = 806) included in the study had a median age of 28 (IQR = 25–31) years. According to the place of residence, 219 (27.2%) came from rural areas and 585 (72.6%) from urban areas, and for 2 (0.2%), the place of provenance was not specified. Ninety-two (11.4%) women were smokers, 13 (1.6%) quit smoking during pregnancy, 673 (83.5%) were non-smokers, and for 28 (3.5%) women the smoking status was unknown. Sixty-four (7.9%) pregnant women had a previous cesarean scar, and the rest did not have any. In the studied group, 62 (7.7%) women had a single previous spontaneous abortion, while 20 (2.49%) had two and 2 (0.25%) women had three previous SAs. Three hundred and forty-one (42.3%) women were experiencing their first pregnancy, 261 (32.4%) their second, 115 (14.3%) their third, 55 (6.8%) their fourth, 18 (2.2%) their fifth, 7 (0.9%) their sixth, 5 (0.6%) their seventh, 2 (0.2%) their eighth, 1 (0.1%) their tenth, and one (0.1%) their thirteenth pregnancy.

Regarding the course of pregnancy, 756 (93.2%) neonates were born from a cephalic presentation and 50 (6.2%) from a breech presentation, 470 (58.3%) women delivered vaginally and 336 (41.7%) women delivered by cesarean section, and 202 women (25.2%) had a spontaneous rupture of the membrane and 601 (74.8%) did not. We have no data regarding the status of spontaneous rupture of membranes for two pregnant women. In addition, 7 (0.4%) women presented placenta previa, 3 (0.4%) women presented abruptio placentae, and 27 (3.3%) presented pregnancy-induced hypertension (PIH).

Six (0.7%) neonates had a birth weight of less than 1500 g, 39 (4.85) had a birth weight of 1500–2499 g, 723 (89.7%) had a birth weight of 2500–3999 g, and 38 (4.7%) had a birth weight of more than 4000 g.

None of the neonates were born at a gestational age below 28 weeks; 7 (0.9%) neonates were born at 29–32 weeks, 11 (1.4%) at 33–34 weeks, 57 (7.1%) at 35–37 weeks, and 731 (90.7%) at 37–43 weeks. The Apgar score at 1 and 5′ was less than 7 for 23 (2.9%) and 6 (0.7%) neonates, between 8 and 9 for 658 (81.6%) and 650 (80.6%) neonates, and between 10 and 61 (7.6%) for 98 (12.2%) neonates. For the rest of the neonates, the Apgar score values at 1 min (n = 52) and 5 min (n = 52) were missed. The gestational age at birth calculated in days was 273 (267–278). For each neonate, the deviation of the true birth weight from the estimated birth weight value was calculated and expressed in MoM. According to this estimation, the value of the birth weight of all neonates from our study expressed in MoM showed a median value of 0.998 [0.923–1.017], which represents a minimal deviation from the estimated birth weight, according to the nomogram by Talge [74].

### 3.2. Latent Cytomegalovirus Infection and the Rate of Previous Spontaneous Abortion

A strong but borderline significant association between the presence of latent CMV infection and the history of SA was found in both situations: if we counted (a) the number of women with spontaneous abortion (total 84) and compared it with the number of women with at least one previous pregnancy (gestation ≥ 2, total 465), and if we counted (b) the total number of spontaneous abortions (total 108) and compared it with the number of all pregnancies that the women had (total 828) in the two groups with and without CMV latent infection (Table 1). 

### 3.3. Association of Latent Cytomegalovirus Infection with Pregnancy Complications and Neonatal Features

Our results did not show any association between the presence of latent CMV infection and the analyzed pregnancy complications (fetal presentation, mode of delivery, prevalence of spontaneous rupture of membranes, abnormal placental insertion, placental abruption, and pregnancy-induced hypertensive disease) or neonatal features (secondary sex ratio, 1′ and 5′ Apgar scores, birth weight, gestational age at birth, length, and head circumference) (Table 2). No difference was found regarding gestational age at birth expressed in days, birthweight expressed in MoM, length, or head circumference between the two groups with and without latent CMV infection.

## 4. Discussion

To our knowledge, the presented study is one of the largest studies worldwide that analyzes the impact of latent CMV infection on a broad range of parameters related to pregnancy and neonatal outcomes or spontaneous abortion history. Moreover, our study allows us to exactly calculate the influence of the presence of latent CMV infection on parameters that are estimated upon exact gestational age calculation (preterm birth, birthweight, etc.). This was possible because the gestational age was measured in each woman according to a first- or second-trimester fetal scan.

Scientific database querying identified only a few studies that analyzed the impact of latent CMV infection on the rate of previous spontaneous abortion [19,49,50,51,54] or pregnancy complications such as pregnancy-induced hypertensive disease [55,56,57,58,60,61,62,63,64,65,66,67], HELLP syndrome [56], IUGR [68], intrauterine fetal death [68], or neonatal features at birth [68].

Previous research shows that along with primary infection, CMV replicates in many cell types, among which are epithelial cells, smooth muscle cells, macrophages, hepatocytes, and vascular cells [22]. Afterward, CMV establishes a lifelong latent infection, and myeloid cells of the bone marrow constitute the virus reservoir [22]. In latent carriers, CMV may occasionally reactivate, causing virus shedding [75,76]. The authors of some studies speculate that reactivation of CMV infection in pregnant women with latent CMV infection could lead to unfavorable neonatal [20] and obstetrical outcomes [77], including SA [50,78]. It is also worth mentioning that children with congenital CMV as a result of maternal non-primary CMV infection can experience congenital or late-onset sequelae such as hearing loss [79].

Latent CMV infection influences T-cell dynamics [28]. Additionally, latent CMV modulates monocyte-mediated innate immune response by stimulating the expansion of macrophage lines, which causes the secretion of proinflammatory cytokines [29] or manipulates the host inflammatory response, which allows for the establishment of latency [31]. Since immune response modulation and inflammation play an important role in the pathogenesis of several processes associated with pregnancy evolution and complications [37,38], we thought it made sense to evaluate whether latent CMV influences the prevalence of pregnancy-associated complications.

Our study showed a higher prevalence of previous SA occurrence in pregnant women with latent CMV infection compared to women without latent CMV infection, but the significance of this difference was borderline (*p* = 0.05). The results in this study are in line with the results of previous studies that found an association between the SA history and the presence of a latent CMV infection [50,51]. Some studies speculate that this association could be explained by the reactivation of latent CMV infection in certain conditions [20,25,75,76], which could cause viremia, infection, damage of the embryo, or miscarriage [53]. Since our study did not analyze the presence of CMV DNA in abortion specimens, we cannot affirm that CMV reinfection was the cause of the previous SA. This is supported by the study of Gao et al., who found that the low CMV DNA prevalence rate in SA specimens and the high anti-CMV IgG seroprevalence suggest that CMV may not be a factor that causes SA in first-trimester pregnancies [52].

Also, our results do not indicate an association between the presence of latent CMV infection and the prevalence of certain pregnancy complications, such as pregnancy-induced hypertension, birth weight, spontaneous rupture of membranes, placental abruption, or placenta previa. Although previous studies showed an association between CMV infection and placental lesions, for example, with the CMV infection potentially causing atherosis in the placentas of women with preeclampsia [80] or disturbing placental vein architectonics [32], not all studies reported a correlation between latent CMV infection and preeclampsia. Thus, studies from some authors argued for an association between latent CMV infection and preeclampsia [55,56,58,60,62,63,65,66], while studies from others did not report the presence of this association [57,58,59,61,64]. In addition to our results, we emphasize that maternal non-primary CMV infection during pregnancy represents the greatest burden [81] because of the high seroprevalence of CMV among women of childbearing age [4]. Therefore, treatment and prevention strategies should be focused not only on primary infection but also on non-primary infection because of the high worldwide prevalence of neurological sequelae secondary to congenital CMV in children born to mothers with pre-existing CMV immunity [81].

A previous study did not find an association between latent CMV infection and low birth weight or IUGR fetuses, which is in concordance with the results of our study [68].

No association was found between the presence of latent CMV infection and other pregnancy complications, such as breech presentation at delivery, cesarean section rate, abnormal placental insertion, duration of pregnancy, placental abruption, or pregnancy-induced arterial hypertension prevalence.

All the neonatal features studied (sex ratio, gestational age at birth, birth weight expressed in grams, and deviation from estimated birth weight expressed in MoM) showed no differences between pregnant women with or without latent CMV infection. Also, our study found that no differences in Apgar score (1 min or 5 min), length, or head circumference existed between neonates in the two groups with and without latent CMV infection.

Only a few studies had been published before and provided data that could be compared with our results. Certain previous research studies argued for the link between the presence of CMV infection and hypertension or cardiac dysfunction [82,83,84]. Most of the previous studies that analyzed the association between the presence of latent CMV infection and pregnancy complications also analyzed the association with the pregnancy-induced hypertension disease and provided controversial conclusions. Although some studies concluded that an association between latent CMV infection and pregnancy-induced hypertension could exist [62,63,77], other studies similar to our study denied the presence of an association [61,64,67].

Some studies analyzed the association between the presence of latent CMV infection and preterm birth, but there is no consensus on the results, as one study argued for the presence of an association [85], while another denied it [45]. Another study analyzed the association of latent CMV infection with the occurrence of low-birth-weight fetuses or neonatal deaths, but the results did not confirm this hypothesis [68]. One of the largest studies was performed by Liu et al. on 18,074 IVF-obtained pregnancies and showed that latent CMV infection did not affect the percentage of women with preterm birth or perinatal death [45].

To our knowledge, there are no studies that have analyzed the association of latent CMV infection and placental insertion, spontaneous rupture of membranes, mode of delivery, placental insertion, placental abruption occurrence, or Apgar score at birth. 

Maternal non-primary CMV infection during pregnancy represents the greatest burden because of the high CMV seroprevalence among women of childbearing age [81]. Therefore, the treatment and prevention strategies should be focused not only on primary infection but also on non-primary infection because of the high worldwide prevalence of neurological sequelae secondary to congenital CMV in children born to mothers with pre-existing CMV immunity [81].

Our study shows some strengths and weaknesses. The main strength of our research is that all the pregnant women in the study are well-characterized regarding gestational age, with an accuracy of a maximum of five days based on the first- or second-trimester scan [69]. This accurate estimation allowed us to evaluate the exact gestational ages and the expected weight of the neonates at birth and further allowed us to study the effect of various factors during the pregnancy period on birth weight and gestational age at birth. Another strength of our research is that a large number of pregnant women were included in the study and that our study is part of a much larger study previously published partially.

The main limitation of our study is that an unbalanced number of women was counted in the two groups with and without latent CMV infection. This could be explained by the high prevalence of CMV infection that exists in the population of our region [4]. Even if the number of patients in the group without latent CMV infection was relatively small, it was enough to evaluate pregnancy complications with a prevalence of 10% such as preterm birth and others. Also, we could not analyze the rate of CMV reactivations in our study or analyze whether the studied pregnancy or neonatal parameters in this research correlated with the anti-CMV IgG titers.

In addition, in our study, no CMV PCR was performed. In a very recently published study, eight commercial immunoassays for the detection of CMV IgM antibodies in pregnancy were analyzed. Until 12 weeks after the onset of primary infection, four assays showed 100% sensitivities, but the remaining had individual gaps to detect all primary infections during this time, which should be considered when interpreting the serology results [86]. A PCR assay would help improve neonatal disease management through timely intervention, especially in cases of asymptomatic infections, enabling early and rapid detection of CMV DNA [87]. Information about the CMV strain and antiviral resistance patterns through the detection of single-nucleotide-level genome modifications are important, since they may have clinical implications [88].

Since we did not measure the CMV viral load in latent infections in our patients, it was not possible to correlate the adverse outcome with this virological indicator. Future studies are needed to analyze the potential impact of viral load in reactivated latent CMV infections as well as in reinfections in CMV-seropositive mothers on pregnancy and perinatal outcomes.

## 5. Conclusions

In conclusion, our results did not show an impact of latent CMV infection on main pregnancy or neonatal outcome features, but the risk of fetal damage due to reactivation of latent infection or reinfection with another CMV strain was not assessed in this study. An important but borderline-significant association was found between the history of previous SA and the presence of latent CMV infection. This latter conclusion should be carefully considered, as the number of women with previous miscarriages and without latent CMV infection was relatively small.

## Figures and Tables

**Table 1:** Association between latent cytomegalovirus infection and history of spontaneous abortion.

		CMV IgG Antibodies				OR [CI]	*p*-Value
		Positive		Negative			
		N	%	N	%		
Obstetrical History							
A—counted as persons (*n* = 465)	Pregnancies	364	81.25%	17	100%	8.467 [0.482–136.37]	0.051
	SA	84	18.75%	0	0%		
B—counted as pregnancies (*n* = 828)	Pregnancies	696	86.57%	24	100%	7.633 [0.460–126.53]	0.061
	SA	108	13.43%	0	0%		

SA = spontaneous abortion, OR = odds ratio, CI = confidence interval.

**Table 2:** Association of latent cytomegalovirus infection with pregnancy complications and neonatal features.

		CMV IgG Antibodies				OR [CI]	*p*-Value
		Negative		Positive			
		N	%	N	%		
Pregnancy Complications							
Fetal presentation	Cephalic	27	84.4%	729	94.2%	0.333 [0.123–0.907]	0.062
	Non-cephalic	5	15.6%	45	5.8%		
Mode of delivery	Vaginal	18	56.3%	452	58.4%	0.916 [0.449–1.868]	0.856
	Cesarean	14	43.7%	322	41.6%		
Spontaneous rupture of membranes	No	23	74.9%	578	74.1%	0.965 [0.425–2.192]	1.000
	Yes	8	25.1%	194	25.9%		
Placental insertion	Normal	32	100%	767	99.1%	1.009 [1.002–1.1016]	1.000
	Previa	0	0.0%	7	0.9%		
Placental abruption	No	32	100%	771	99.6%	1.004 [0.999–1.008]	1.000
	Yes	0	0%	3	0.40%		
Pregnancy-induced hypertension	No	31	96.9%	748	96.6%	1.078 [0.142–8.199]	1.000
	Yes	1	3.1%	26	3.4%		
Neonatal Features							
Secondary sex ratio	Girls	15	46.9%	387	50.0%	0.882 [0.434–1.762]	0.857
	Boys	17	53.1%	387	50.0%		
Apgar 1′	<7	1	3.3%	22	3.1%	1.965 [0.21–17.19]	0.225
	8–9	24	80%	634	89%	2.359 [0.866–6.42]	
	10	5	16.7%	56	7.9%	R	
Apgar 5′	<7	0	0%	6	0.8%	278 [44.5–1740]	0.765
	8–9	24	82.8%	626	86.3%	1.391 [0.51–3.74]	
	10	5	17.2%	93	12.8%	R	
Birth weight categories (grams)	≤1500	0	0.0%	6	0.8%	1.076 [0.175–6.619]	0.365
	1501–2500	0	0.0%	38	5.0%	29.872 [2.155–40.128]	
	2501–4000	30	93.8%	693	89.5%	R	
	>4000	2	6.2%	36	4.7%	1.526 [0.793–2.936]	
Gestational age at birth categories (weeks)	<32	0	0.0%	7	0.87%	3226.33 [2.21 × 10^−84^–4.71 × 10^+90^]	0.434
	32–33	0	0.0%	11	1.36%	707.97 [1.89 × 10^−30^–2.66× 10^+35^]	
	34–36	1	0.12%	56	6.95%	2.46 [0.33–18.53]	
	37–39	24	2.98%	546	67.74%	R	
	≥40	7	0.87%		20.4%	0.967 [0.41–2.29]	
		Median (IQR)		Median (IQR)			
Gestational age (days)		274 * (266–280)		273 * (267–278)			0.818
Birth weight (MoM)		1.021 * (0.918–1.135)		0.998 * (0.923–1.075)			0.413
Length (cm)		51 * (50–52)		50 * (49–52)			0.136
Head circumference (cm)		34 * (33–35)		34 * (33–35)			0.149

* Median (IQR), R = reference, OR = odds ratio.

## Data Availability

The data sets used and/or analyzed during the present study are available from the first correspondence author upon reasonable request.
